# Vaccine Efficacy and Safety in Patients with Celiac Disease

**DOI:** 10.3390/vaccines12121328

**Published:** 2024-11-26

**Authors:** Rocco Scarmozzino, Giovanna Zanoni, Alessandra Arcolaci, Rachele Ciccocioppo

**Affiliations:** 1 Immunology Unit, Azienda Ospedaliera Universitaria Integrata Policlinico G.B. Rossi & University of Verona, 37134 Verona, Italy; alessandra.arcolaci@aovr.veneto.it; 2 Gastroenterology Unit, Department of Medicine, Azienda Ospedaliera Universitaria Integrata Policlinico G.B. Rossi & University of Verona, 37134 Verona, Italy; rachele.ciccocioppo@univr.it

**Keywords:** celiac disease, efficacy, hyposplenism, immunization, infections, safety, vaccines

## Abstract

Celiac disease (CD) is an autoimmune disorder caused by gluten intake in genetically predisposed individuals. This article provides an overview of the available data on the risks of infectious diseases and the mechanisms involved in CD, including a detailed analysis of vaccine efficacy, immunogenicity, and safety. The published articles were retrieved from the PubMed database using the terms “celiac disease”, “efficacy”, “hyposplenism”, “immune response”, “infections”, “immunization”, “immunogenicity”, “safety”, “vaccination”, and “vaccine”. CD can be associated with several autoimmune diseases, including selective immunoglobulin A deficiency (SIgAD), altered mucosal permeability, and hyposplenism. These conditions entail an increased risk of infections, which can be prevented by targeted vaccinations, although specific recommendations on immunization practices for subjects with CD have not been released. Regarding vaccinations, the immune response to the *Hepatitis B virus* (HBV) vaccine can be impaired in patients with CD; therefore, proposed strategies to elicit and maintain protective specific antibody titers are summarized. For patients with conditions that put them at risk of infections, vaccinations against *Pneumococcus* and other encapsulated bacteria should be recommended. Based on the available evidence, the *Rotavirus* vaccine offered to children could be useful in preventing CD in at-risk subjects. Overall, except for the HBV vaccine, vaccine efficacy in patients with CD is comparable to that in the general population, and no safety concerns have arisen.

## 1. Introduction

Celiac disease is an autoimmune disorder caused by gluten intake in genetically predisposed subjects; the disease may develop at any age, with a highly polymorphic clinical presentation [1]. The diagnostic hallmarks of the disease are the presence of serum anti-transglutaminase autoantibodies and different degrees of villous atrophy and inflammatory infiltrate upon histologic examination of the duodenal mucosa in active disease, which revert upon the establishment of a gluten-free diet (GFD), which is the only effective therapy [1]. The worldwide prevalence of CD is estimated to be 0.6–1.0% in the general population [2], whereas it is higher in patients with further immune-mediated diseases [3,4], such as type 1 diabetes (T1D) [5], Hashimoto’s thyroiditis [6], selective immunoglobulin A deficiency (SIgAD) [4], and several others [7,8]. Furthermore, patients with CD carry an increased risk of serious infections, such as pneumococcal pneumonia, and mortality [3,9,10].

It is well recognized that vaccines play a pivotal role in primary prevention against infectious diseases, considerably reducing morbidity and mortality. However, the immune response to vaccines can be influenced by several factors, such as age, sex, ethnicity, genetic variation, immune competence, psychological stress, nutrition, and vaccine characteristics [11].

Patients at an increased risk of infection complications, such as sepsis, should undergo an immunological evaluation and be offered personalized immunization schedules to achieve the best level of protection against vaccine-preventable diseases.

This review aims to provide an updated overview of the data published in the literature regarding immunological issues relevant to the defense against infections and the response to vaccination in patients with CD. It also presents suggestions to guide adequate immunization in clinical practice.

### Methods

For this narrative review, an extensive literature search of articles in English and Italian within the PubMed Medline database published between January 1981 and June 2024 was carried out using combinations of the following terms as search keywords: “celiac disease”, “hyposplenism”, “immunological response”, “infections”, “immunization”, “vaccination”, “vaccine”, “efficacy”, “immunogenicity”, and “safety”. Papers have been screened for relevance. Randomized controlled studies, observational studies, reviews, meta-analyses, and guidelines regarding pediatric and adult populations were included. We excluded studies in other languages and studies of animal models. 

## 2. Risk of Infections and Immunologic Dysfunction in Celiac Disease

Increased susceptibility to infections has been shown in patients with CD [12,13], mostly caused by encapsulated bacteria, such as *Streptococcus pneumoniae*, *Neisseria meningitidis*, and *Haemophilus influenzae*, with a high risk of sepsis and fatal outcomes [14,15,16,17,18]. Moreover, the risk of further infectious diseases, including influenza [19], tuberculosis [20], and *Clostridium difficilis* colitis [21], has also been found to be increased. The heightened susceptibility to infections in patients with CD likely arises from multiple factors, which are analyzed below.

### 2.1. Hyposplenism

Splenic hypofunction/atrophy is the main cause of the increased risk of infections in patients with CD, and it has been reported in 20–80% of patients, depending on the method applied in each study’s evaluation [13,22,23,24]; this condition is most frequent in those patients who received a diagnosis of CD in adulthood [25,26], especially in those suffering from further autoimmune disorders or with refractory CD (i.e., a severe malabsorption syndrome that is non-responsive to a gluten-free diet (GFD) in the absence of immunodeficiency). In addition, a correlation between splenic atrophy and the occurrence of mesenteric lymph node cavitation (MLNC) syndrome and enteropathy-associated T cell lymphoma has been described, with both complications carrying a poor prognosis [27,28]. This is why hyposplenism is recognized as an indicator of poor prognosis in adult patients with CD. However, the establishment of a GFD reverts splenic function, except for in those patients with splenic atrophy [27,29], which is a permanent condition that is deserving of an appropriate vaccination schedule, similar to that for subjects who undergo a splenectomy [22].

The histologic features are characterized by severe atrophy of the marginal zone of the white pulp and a marked reduction in the expression of IgM memory B cells [30], a population that carries the ability to produce natural antibodies, including those directed against *Streptococcus pneumoniae*, *Neisseria meningitidis*, and *Haemophilus influenzae* [22,31,32], as well as the ability to initiate T cell-independent immune responses upon vaccination with capsular polysaccharide antigens [25]. In addition, a defective opsonization function that occurs due to a reduction in tuftsin activity contributes to the increased susceptibility to infections, while a reduction in regulatory T cells is thought to be responsible for the increased susceptibility to autoimmune phenomena [22,33,34].

Another pivotal role of the spleen is to clear the blood from senescent red cells, a function that is anatomically localized to the red pulp. Its impairment results in the defective removal of pits from erythrocytes, with a consequent increase in circulating pitted red cells and Howell–Jolly bodies and a reduction in platelet sequestration, resulting in thrombocytosis, thus increasing the risk of thromboembolism [35,36,37].

Defective spleen function is believed to stem from the following mechanisms: reticuloendothelial atrophy caused by lymphocyte depletion or the failure of splenic recirculation due to loss or their trapping in the gut, reticuloendothelial blockade secondary to circulating immune complexes or pathogens, and autoimmune damage, leading to the atrophy of the marginal zone of the white pulp where IgM memory B cells reside [27].

Splenic hypofunction or atrophy can be identified by counting Howell–Jolly bodies or pitted erythrocytes through phase-interference microscopy in peripheral blood smears [16,38]. More recent methods include assessing circulating IgM memory B cells through flow cytometry and using nuclear imaging techniques with radio-isotopic elements [32,39]. Additionally, spleen atrophy can be easily detected through abdominal echotomography.

The frequent occurrence of splenic hypofunction and its dangerous consequences emphasize the importance of evaluating splenic function in patients diagnosed with CD in adulthood, those with concurrent autoimmune diseases or a history of significant infections/sepsis or thromboembolism [34,36,40], or those with evidence of MLNC [36]. The British Society of Gastroenterology [41], along with other authors [34,36], recommends that adults with CD receive vaccinations with protein-conjugated vaccines (PCVs) against *Streptococcus pneumoniae* [37].

Due to the increased risk of serious infections by encapsulated microorganisms in individuals with splenic hypofunction, it is also recommended that subjects with CD and associated hyposplenism receive immunization against *H. influenzae type b* and *N. meningitidis* using a tetravalent conjugate ACWY vaccine [1,32,42,43].

### 2.2. Altered Mucosal Permeability

A defective gut barrier is involved in both the pathogenesis of CD and the increased risk of infections due to heightened intestinal permeability [44]. The latter also depends on an imbalance between enterocyte apoptosis and proliferation, resulting in the intestinal mucosa being covered by immature cells with immature junctional complexes [45]. Additionally, nutritional deficiencies, particularly in folate and vitamin B12, may impact both enterokinetics and immune competence. In this regard, the frequent poor vitamin D status observed in patients with CD may contribute to a greater burden of respiratory infections, including influenza and tuberculosis [35].

### 2.3. Selective IgA Deficiency

SIgAD is observed at a higher rate in patients with CD compared to the general population, with a prevalence of approximately 2.0–2.5% [21]. Studies conducted in children indicate a prevalence of up to 10% of CD in SIgAD patients [46]. The majority of individuals with SIgAD are asymptomatic due to immunologic compensatory mechanisms that may prevent infectious manifestations. However, some patients develop recurrent infections of the respiratory and/or gastrointestinal tract [47] because secretory IgAs are a component of the gut and respiratory barriers, where they prevent microorganisms from adhering to the mucosa. Moreover, SIgAD could trigger a dysregulated immune response, leading to an increased prevalence of concurrent autoimmune diseases [48] that, in the case of individuals genetically predisposed to CD, may contribute to the development of an immune response against gluten-derived peptides [46].

Additionally, IgAs are likely involved in maintaining overall immune system balance. Indeed, lower regulatory T cells have been observed in SIgAD patients, along with various alterations in memory B cells, all of which could potentially contribute to autoimmunity [46]. Several factors and mechanisms could be implicated in the connection between CD and SIgAD. These include the human leukocyte antigen (HLA) system, particularly DQ2-related allelic variants, non-HLA genes, and environmental factors [46]. It is well recognized that CD is closely related to the presence of HLA-DQ2 and/or DQ8 heterodimers, and included among the haplotypes mostly associated with SIgAD are also allelic variants encoding for the HLA-DQ2 heterodimer [49]. Therefore, HLA-DQ genes may simultaneously contribute to the development of SIgAD and predispose an individual to CD [46]. In addition, recent evidence supports the hypothesis of the involvement of non-HLA genes and/or epigenetic influences from environmental factors in the development of SIgAD, such as dietary gluten exposure and intestinal dysbiosis [46].

## 3. Immune Response to Vaccines in Patients with Celiac Disease

Immunization is one of the most effective measures of the public health system, saving countless lives and preventing lifelong disabilities [50]. The vast majority of vaccines are extremely effective and highly safe. Therefore, they have not only led to a reduction in the incidence of infections, but also in related mortality and morbidity [50]. Of course, vaccinations are particularly important for individuals at increased risk of complications due to their age, underlying disease, or treatments impairing the host immune response.

Literature data on the efficacy and immunogenicity of vaccines in individuals with autoimmune diseases are available [51]; however, studies regarding CD are limited. The most studied issue focuses on the immunological response to the *Hepatitis B virus* (HBV) vaccine, while few available data regarding other vaccinations are reported.

### 3.1. Hepatitis B Vaccination

The recombinant HBV vaccine induces a protective immune response in approximately 95% of healthy individuals [52,53]. Non-responsiveness, defined as the inability to generate a protective level of HbsAb (≥10 mU/mL), has been linked to various conditions and underlying diseases, such as dialysis, obesity, age, male gender, HIV infection, and type 1 diabetes mellitus [11,54,55,56,57]. It has also been associated with the presence of the HLA haplotypes HLA DR3, DR7, and DQ2 [58,59,60,61]. The strongest correlation with an inadequate immune response to the vaccine has been shown with HLA-DQ2 molecules, which are found in nearly 40% of the Caucasian general population and in approximately 90% of patients with CD [3].

The pathogenetic mechanism of the reduced humoral response to HBV in CD involves the competition between HBsAg protein fragments and gliadin peptides for binding to HLA-DQ2 molecules, causing defective antibody production against HBsAg in active CD [62]. Therefore, the presentation of HBsAg can occur with low affinity by specific HLA serotype groups in these genetically predisposed individuals [58].

A meta-analysis published in 2015 showed that patients with CD have a statistically significant lower rate of protective HBsAb titer compared to non-affected controls [56]. These data were confirmed by similar results found in other studies [62,63,64,65,66,67]. Consequently, a large number of patients with CD may be non-responders to HBV vaccination [56,68]. Similarly, a defective response to the HBV vaccine has also been reported in type 1 diabetes mellitus (T1DM), which has approximately 90% of HLA alleles in common with CD [69,70].

An inverse correlation exists between the levels of anti-HBs antibodies and the time interval from vaccination to sample collection for HBsAb determination in healthy subjects [71]. However, the decline in HBsAb titer over time may be faster in patients with CD until it becomes undetectable in 15–50% of cases within 5 to 10 years [56,70]. Therefore, it is important to routinely assess the serologic response to HBV in newly diagnosed CD patients who have previously been vaccinated [69].

Several studies have examined whether active CD and a GFD affect the achievement of protective HBsAb titers. Nemes et al. compared the seroconversion rate after HBV vaccination in children and adolescents with a confirmed CD diagnosis under a strict GFD at the time of vaccination and those not yet diagnosed on a gluten-containing diet [62]. They found a significantly higher rate of non-responders (74.1%) in the undiagnosed group exposed to gluten, suggesting a link between a lower response to HBV vaccination, disease activity, and gluten intake. More recently, Trovato et al. reported that elevated IgA anti-transglutaminase levels, markers of active CD, and an older age at diagnosis in children were associated with a lack of seroconversion to the HBV vaccine [72]. Similarly, Ertem et al. found that the serological response to the HBV vaccine in children who adhered to a GFD was comparable to that of a healthy population and that non-responder status is not permanent, as compliant treatment with a GFD for at least one year can improve the immune response to the HBV vaccine [64].

The beneficial effect of a GFD was not supported in the retrospective study of Zifman et al., who found that, in previously vaccinated pediatric subjects with active CD, a GFD did not enhance the rates of HBsAb concentrations [73]. Similarly, Zingone et al. showed a higher rate of inadequate response to the HBV vaccine in children and young adult celiac patients vaccinated as infants on a GFD compared to healthy controls, suggesting a more prominent role of immunological factors and/or genetics rather than gluten exposure alone [74].

The reduced immune response to HBV vaccination represents a reservoir of people susceptible to HBV infection and, therefore, at risk of contracting and spreading the disease [69]. Consequently, targeted vaccination strategies to achieve full protection against HBV, such as revaccination, different immunization schedules, alternative routes of administration, and special vaccine formulations, have been evaluated. In this regard, a suggested strategy to improve HBV vaccine efficacy consists of administering booster doses or repeating vaccination of low- and non-responders to increase the response rate [75]. Rousseff evaluated the response to a booster HBV vaccination in previously vaccinated non-responder children with CD, concluding that a single intramuscular booster is able to induce a serological response in two-thirds of the initial non-responders [76]. In another study, similar immunization rates in celiac patients were achieved after the first booster dose, with an even higher response rate (96.4%) after three booster doses [77]. Vitaliti et al. proposed that all patients with CD should receive a booster dose of the HBV vaccine every 10 years during disease remission, regardless of their unresponsiveness status, as it would be a better strategy to maintain full protection from HBV infection [78]. Moreover, Balamtekın et al. compared two different HBV immunization protocols for children with CD: 0, 2, and 9–12 months of life or 0-, 1-, and 6-month schedules. The response rates, evaluated by HBsAb determination, did not show any statistically significant differences between the two schedules in terms of protection [65].

Further studies have aimed to analyze if the serological response to the HBV vaccine differs when using the intradermal (ID) route as an alternative to the conventional intramuscular (IM) administration [79,80,81,82]. The rationale behind this is that the skin is a more immunogenic site than the muscle due to the presence of dendritic cells in the dermis, which are capable of presenting antigens and stimulating innate and adaptive immune responses [79]. In patients undergoing hemodialysis, who have a diminished humoral response due to alterations in T lymphocytes and antigen-presenting cells, a higher seroprotection rate was observed after ID vaccination compared to IM vaccination [80,81]. A systematic review with a meta-analysis analyzed the levels of seroprotection achieved by the HBV vaccine when administered intradermally compared to intramuscularly [82], finding that while ID vaccination was associated with slightly lower protective antibody levels overall than the IM route, there were only marginal differences in seroprotection in most studies. Leonardi et al. compared the ID and IM routes for the HBV booster vaccine in celiac children vaccinated during their first year of life with an absence of protective HBsAb levels. They found that both routes are effective in stimulating the production of HBV antibodies with a similar percentage of responders; however, the ID route showed a greater proportion (40%) of high responders (anti-HBs > 1000 IU/L) compared to the IM route (7.1%) [77]. These data demonstrate comparable immunization rates between the two routes, suggesting that the ID route could be a valid alternative to IM vaccines. An advantage of the ID route could also be its better cost/benefit ratio, as it requires a lower vaccine dose; additionally, HBsAb determination may be unnecessary since cellular immune responsiveness to HbsAg can be assessed by evaluating cutaneous reaction at the injection site (Table 1) [77,79].

Lastly, a special formulation of the HBV vaccine containing recombinant HBsAg plus Pre-S/S epitopes showed higher immunogenicity compared to standard vaccines in high-risk patients, including those who do not respond to conventional HBV vaccines [83]. In this regard, Heshin-Bekenstein compared the short-term immunogenicity of a Pre-S-containing vaccine with a conventional formulation in previously immunized patients with CD and non-protective HBsAb concentrations [84]. Both vaccines elicited adequate responses following a single injection (98% for the Pre-S vaccine and 87% for the conventional vaccine); however, the Pre-S-containing vaccine resulted in higher HBsAb concentrations. Non-responders received two more doses, and they all showed seroprotective levels after the third dose.

### 3.2. Pneumococcal Vaccination

Two types of pneumococcal vaccines are currently available: pneumococcal conjugate vaccines (PCVs)—either 13-valent (PCV-13), 15-valent (PCV-15), or 20-valent (PCV-20)—and polysaccharide 23-valent vaccines (PPV-23). PCVs contain a carrier protein, typically a modified diphtheria toxin named CRM197, which elicits a T-dependent immune response [85], while PPV-23 provides protection through a T-independent mechanism. The primary series of PCV is recommended for all children under two years of age in most countries, while PPV-23 is used in combination with PCV for certain high-risk groups and in the elderly to ensure protection against a broader spectrum of serotypes [86,87]. The implementation of PCV in mass vaccination programs has led to a substantial decrease in the incidence of pneumococcal diseases among both vaccinated and unvaccinated individuals of all ages [88]. In contrast, several studies have shown that patients with CD have a higher rate of pneumococcal infections when compared to healthy controls [16,45,89], with hyposplenism being identified as the main cause of increased susceptibility to pneumonia and sepsis [16,17,90].

Pneumococcal conjugate vaccination elicits a T cell-dependent immune response, allowing for the restoration of the pool of pneumococcal polysaccharide-specific memory B lymphocytes that have been found to be reduced in asplenic individuals [91].

Pneumococcal vaccination is currently recommended for all patients with functional hyposplenism [31,34,43,92], including the form associated with CD (Table 1).

A retrospective analysis of an English cohort under the age of 65 years found that the overall absolute rate of pneumonia was similar in patients with CD and the controls; however, unvaccinated celiac patients showed a 28% higher risk of pneumonia compared to healthy unvaccinated controls [14], indicating the importance of immunization for this population. Another study confirmed the importance of pneumococcal vaccination in patients with CD by comparing the rate ratio of pneumonia in celiac patients between two populations: the first population comprised data from 1963 to 1999, representing a pre-vaccination era, and the second population incorporated data from 1998 to 2003, a period when pneumococcal vaccine availability was widespread. The results showed an increased rate of pneumonia in the pre-vaccination era population [16].

Moscatelli et al. examined immune responses in patients with active and treated CD induced by the administration of the PCV-13 vaccine—a T-dependent stimulation—and the PPV-23 vaccine—a T-independent stimulation—through the assessment of serotype-specific IgG levels and using an opsonophagocytic assay to determine the functional ability to opsonize and promote the phagocytosis of *pneumococcus* [31]. Both vaccines were found to be immunogenic, but PCV-13 provided higher and more sustained protection against clinically relevant serotypes associated with a higher risk of pneumonia and mortality compared to that generated by PPV-23. Additionally, they demonstrated a high frequency of functional B cell defects in both treated and untreated CD, indicating that all celiac patients should be considered at high risk of pneumococcal infection, independent of the presence of hyposplenism and supporting immunization programs that provide vaccinations to risk groups [31].

### 3.3. Other Vaccinations

The antibody response to vaccines in patients with CD has been found to be comparable to that of the general population, with the exception of HBV [61,93,94].

Park et al. evaluated the serologic response to tetanus, rubella, and *Haemophilus influenzae type b* (Hib) vaccines in children with CD and healthy controls, showing similar protective titers between the two groups [68].

Comparable results were obtained from an Italian study that showed no significant differences in the humoral immune response to vaccines against poliomyelitis, diphtheria, tetanus, measles, mumps, rubella, and pertussis between children with CD and the controls [95].

Even in another study, no statistically significant difference was found in the serological responses to one dose of measles-containing vaccines in patients with CD, T1DM, and the controls [70]. Furthermore, Urganci and Kalyoncu evaluated the response to HAV and HBV vaccines in 16 pediatric patients with CD and 35 controls; they found that after the HAV vaccine, protective antibodies developed in 75% of individuals with CD compared to 100% of healthy controls [66]. Interestingly, subjects who were unresponsive to the HAV vaccine also showed non-responsiveness to the HBV vaccine.

Different data emerged from the study reported by Sari et al. regarding 33 children with CD and 62 healthy controls [96]. At one month after vaccination, seroconversion rates to HAV were 78.8% in children with CD and 77.4% in the controls, and an increase in the rate of seroconversion to 97% and 98.4% was observed at seven months. This is in contrast with the results of Urganci et al., which showed no change in response rates during a seven-year follow-up period [66]. The sample size of these studies is limited, and further research is required for final assessments.

At the beginning of the COVID-19 pandemic, concerns arose regarding a potential increased risk of contracting or experiencing a more severe *SARS-CoV-2* infection in the CD population [97]. Additionally, there were initial apprehensions about a possible diminished response to the vaccine in individuals with CD. However, studies have revealed that patients with CD do not face an elevated risk of contracting the virus or experiencing a severe course of COVID-19 compared to the general population [98,99,100,101]. Regarding the immune response to COVID-19 vaccines, some studies on patients with CD vaccinated with viral vector or mRNA formulations have shown that humoral responses targeting the spike protein of *SARS-CoV-2* were comparable to those of healthy controls [102,103].

## 4. Vaccine Safety

Vaccines have long been suspected to be a trigger for the development of autoimmune diseases [104]. The main mechanism proposed to explain this association is epitope mimicry, where vaccine antigens share structural similarities with self-antigens, similar to what may occur during infections. The immune response to the vaccine antigen might, therefore, also extend to other host cells expressing the structurally similar self-antigen. A second mechanism that could be involved is bystander activation, which leads to the activation of autoreactive T cells [105,106]. However, the pathogenetic mechanisms that could explain the causal link between vaccinations and autoimmune diseases are not yet fully understood and are also difficult to study [107].

In the scientific literature, many isolated cases or case series of autoimmune disorders or the reactivation of pre-existing diseases have been reported as potential vaccine adverse events. Nevertheless, epidemiological studies do not support the hypothesis that vaccinations can cause autoimmune diseases [104,105,106,107,108]. In this regard, a retrospective study assessing a possible connection between measles vaccination and the development of CD, Crohn’s disease, and ulcerative colitis did not demonstrate significant differences in the prevalence of both CD and inflammatory bowel diseases between the vaccinated and unvaccinated groups [109].

Regarding a potential increase in risk due to different vaccination schedules, a study investigated whether changes in the national vaccination calendar correlated with changes in the incidence of CD during the “Swedish CD epidemic” from 1984 to 1996 [110]. Another study compared the cumulative incidence of CD between Estonian and Finnish children to determine if changes in vaccination programs between the two countries could be associated with changes in CD incidence [111]. In both studies, the authors concluded that modifications in the vaccination schedule, including the types of vaccines, age at administration, and number of doses, were not associated with an increased risk of developing CD [110,111]. Interestingly, the *Bacillus Calmette–Guérin* (BCG) vaccination appeared to have a protective effect, as it reduced the risk of developing CD [110].

Finally, considering that gastrointestinal infections play a role in the pathogenesis of CD [112], concerns were raised about the possibility that the live oral *rotavirus* (RV) vaccine could cause structural and functional lesions in the upper small intestine, thus increasing the risk of developing CD. However, Hemming-Harlo et al. found that the live RV vaccine induces a subclinical infection without causing gastroenteritis, which may not be sufficient to trigger CD. Therefore, intestinal damage has only been associated with wild-type RV infection [113]. Similarly, Vaarala et al. carried out a cohort study to evaluate whether RV vaccination affects the risk of CD, and their results showed no significant differences in the incidence of CD between the group vaccinated against RV and unvaccinated children with follow-up for four to six years [114]. Remarkably, an initial trend emerged, indicating a lower incidence of CD in the vaccinated group. This difference became more evident over a longer follow-up period; indeed, Hemming-Harlo et al. extended the study to 11–14 years and found a significant reduction in the risk of CD autoimmunity in children vaccinated with an RV vaccine, suggesting its protective role in the early years of life (Table 1) [113]. A recent meta-analysis further confirmed the absence of the additional risk of triggering CD or T1DM in children vaccinated against *rotavirus* [115].

**Table 1:** Summary of findings and suggested strategies for vaccination of subjects with celiac disease.

Vaccines	Target Population	Findings	Suggested Strategies
Hepatitis B	Children Adults	− HLA-DQ2-positive subjects with CD may have an impaired response (HBsAb < 10 mU/mL) [58,59,61] − Impaired response observed in subjects vaccinated during active CD [62,64,72] − Faster decline of HBsAb titers in CD [56,70]	Check HBsAb titer in subjects vaccinated before CD diagnosis [69] If HBsAb < 10, administer a booster dose of vaccine and check HbsAb titer after 1 month [75,76] If HBsAb < 10, administer a booster dose of vaccine and check HbsAb titer after 1 month [75,76] Administer up to three doses to non-responders to one dose [77] Administer HBV vaccine during clinical remission of CD [64,78] Administer a booster dose of HBV vaccine every 10 years during disease remission, regardless of their responsiveness status [78] Both intradermal and intramuscular routes are effective in stimulating the production of HBV antibodies [77]
Pneumococcal vaccine	Children Adults	− Subjects with CD and hyposplenism are at an increased risk of invasive infections (pneumonia and septicemia) [14,16,17,18,41] − The immune response is similar to that of healthy controls [68] − Both the PCV and PPV-23 vaccines are immunogenic, although PCV provides higher protection against higher-risk serotypes [31]	Evaluate splenic function in subjects at risk [22,32,36] Recommend administration of the pneumococcal conjugated vaccine [31,37]
Rotavirus	Children	− Rotavirus infection in early life has been implicated in the development of CD [112,113] − Rotavirus vaccination could potentially prevent the development of CD [113,114,115]	Consider RV vaccination to individuals at risk of developing CD [113,114]
Haemophilus influenzae type B	Children Adults	− Patients with hyposplenism have an increased susceptibility to infections by encapsulated bacteria [15,37] − The immune response to the HiB vaccine is similar to that of healthy controls [68]	Recommend HiB vaccination for subjects with CD and associated hyposplenism [32,42,43]
Neisseria meningitidis	Children Adults	− Patients with hyposplenism have an increased susceptibility to infections by encapsulated bacteria [15,37] − The immune response to the HiB vaccine is similar to that of healthy controls [68]	Recommend meningococcal ACWY vaccination for subjects with CD and associated hyposplenism [32,43]
Hepatitis A	Children	− Normal or reduced immunogenicity in subjects with CD [66,96]	Further research is required for final assessment
Measles, Mumps, Rubella	Children Adults	− The immune response is similar to that of healthy controls [70,95]	Recommendations as per the national immunization program
Diphtheria, Tetanus, Pertussis, Polio	Children	− The immune response similar to that of healthy controls [95]	Recommendations as per the national immunization program
SARS-CoV-2	Children Adults	− No increased risk of severe COVID-19 in CD compared to the general population [98,99,100,101] − The immune response to the vaccine is similar to that of healthy controls [102,103]	Recommendations as per the national immunization program

## 5. Attitude Toward Vaccinations Among Patients with CD

Although vaccines and vaccination procedures are currently extremely safe, concerns regarding vaccine safety and the risk of potential adverse reactions represent a complex global phenomenon and a significant public health challenge [116,117,118].

A recent Italian survey analyzed data on 130 patients with CD who answered a web-based questionnaire [119]. The results showed that while the majority of patients exhibited a strongly positive attitude toward vaccinations, a significant minority (one out of five) held a partially negative stance. Three-quarters of the CD patients could remember their vaccination status, but only a small percentage (16–20%) indicated that they had received vaccinations against certain vaccine-preventable diseases, such as meningitis and *pneumococcus*, which could pose a threat to their health. Similarly, Rehman et al. conducted an online survey completed by 99 CD patients, estimating that 76% expressed a favorable attitude toward vaccinations [120]. They analyzed determinants that significantly influenced positive attitudes, including higher education levels, concerns about the potential recurrence of vaccine-preventable diseases (VPDs), and confidence in healthcare experts compared to mass media. Conversely, engaging in complementary and alternative medicines, expressing the desire to postpone vaccination, and having previous personal or relatives’ negative experiences with vaccinations were factors associated with a significantly negative disposition.

Regarding the COVID-19 vaccine, a survey conducted in Italy revealed that nearly 25% of individuals with CD expressed hesitancy toward vaccination, with a refusal rate of 4.8%, primarily due to concerns about potential adverse events and the accelerated vaccine development. Notably, 27% of hesitant individuals attributed their concerns about vaccine efficacy and safety to being influenced by CD. Only a minority (3%) believed that having CD increased the risk of COVID-19 and were thus willing to prioritize vaccination [121].

Optimizing communication between patients and physicians plays a fundamental role in the management of patients who need to undergo immunization, improving awareness of the perceived versus real risks related to vaccination, as well as the importance of being vaccinated against preventable infectious diseases.

## 6. Conclusions

The available evidence suggests that there are no safety concerns regarding any vaccination in children with CD, and overall, the immunogenicity of vaccines is comparable to that in the general population, except for the response to the HBV vaccine.

Most evidence links the defective response to the HBV vaccine to specific HLA haplotypes, while others propose that active CD with gluten exposure at the time of vaccination may also be a contributing factor.

It is recommended that HBsAb titers be routinely evaluated in individuals with newly diagnosed CD who have previously been vaccinated. In cases of non-protective antibody levels, it is recommended to receive a booster dose or a complete three-dose series of the HBV vaccine, along with a subsequent follow-up of serological protection. Interestingly, some studies have reported that RV vaccination may play a protective role in preventing the development of CD.

Due to the association between adulthood CD and splenic hypofunction, which predisposes individuals to serious infections by encapsulated microorganisms, especially in those with concomitant autoimmune diseases and/or refractory CD, immunization against *pneumococcus, H. influenzae type b,* and *N. meningitidis* with the tetravalent conjugate ACWY vaccine is recommended.

In conclusion, no vaccine safety or efficacy concerns have arisen in patients with CD, except for the HBV vaccine, where several studies have demonstrated a higher rate of hypo-responders.
